# Species-Specific qPCR Detection Reveals Offshore Distribution of *Gonyaulax polygramma* (Dinophyceae) in Korean Coastal Waters

**DOI:** 10.3390/biology15131048

**Published:** 2026-07-01

**Authors:** Jinyeong Jung, SeoYeol Choi, Seok Hyun Youn, Seok Jin Oh, Tae Gyu Park

**Affiliations:** 1 National Institute of Fisheries Science, Busan 46083, Republic of Korea; dinophysis@korea.kr (J.J.); seoyeol@korea.kr (S.C.); younsh@korea.kr (S.H.Y.); 2 Department of Oceanography, Division of Earth Environmental System Science, Pukyong National University, Busan 48513, Republic of Korea; sjoh1972@pknu.ac.kr

**Keywords:** *Gonyaulax polygramma*, *Gonyaulax*, dinoflagellate, qPCR, molecular detection, species-specific assay, harmful algal blooms

## Abstract

*Gonyaulax polygramma* is a bloom-forming dinoflagellate that can be difficult to identify accurately during routine harmful algal bloom (HAB) monitoring, particularly when morphologically similar *Gonyaulax* species occur together in field samples. To improve species discrimination and quantitative detection, we developed a species-specific TaqMan qPCR assay targeting the 28S rDNA region of *G*. *polygramma* and applied it to field samples collected during a four-year survey in Korean coastal waters. The developed primer–probe set showed high specificity without cross-reactivity with tested non-target microalgae. The assay exhibited high linearity (R^2^ = 0.999), amplification efficiencies of 92.01–99.36%, and a detection limit of ${10\;copies\;reaction}^{-1}$. Field application revealed clear regional differences in distribution patterns. *G*. *polygramma* was rarely detected in the semi-enclosed Jinhae Bay, whereas relatively high abundances were observed at offshore-influenced stations in the Tongyeong–Yeosu–Wando region during summer. These results suggest that *G*. *polygramma* occurs locally in offshore-influenced coastal waters rather than being uniformly distributed along the southern coast of Korea. The developed qPCR assay provides a reliable molecular tool for detecting *G*. *polygramma* and can complement morphology-based monitoring of *Gonyaulax* species in coastal HAB surveillance programs.

## Simple Summary

Morphological similarity among dinoflagellate species can lead to misidentification during harmful algal bloom (HAB) monitoring. *Gonyaulax polygramma* is not currently recognized as a toxin-producing species, but high-density blooms of this species can cause water discoloration, oxygen depletion, and ecological disturbances in coastal waters. In addition, some morphologically similar *Gonyaulax* species, such as *Gonyaulax spinifera*, have been reported to produce yessotoxins, indicating the need for accurate species-level identification within this genus. To improve the detection and quantification of *G*. *polygramma*, we developed a species-specific TaqMan qPCR assay and applied it to Korean coastal waters over a four-year survey period. The results showed that *G*. *polygramma* was rarely detected in the semi-enclosed Jinhae Bay, whereas relatively higher abundances were observed in the offshore Tongyeong–Yeosu–Wando coastal region during summer. The qPCR assay developed in this study provides a useful molecular tool for distinguishing *G*. *polygramma* from morphologically similar *Gonyaulax* species and improves the reliability of HAB monitoring in aquaculture-intensive coastal waters.

## 1. Introduction

*Gonyaulax polygramma* is a marine dinoflagellate known to form high-density blooms in coastal waters, particularly during summer. This species exhibits a mixotrophic nutritional strategy, combining photosynthesis with prey ingestion, which may contribute to its survival and growth under varying environmental conditions [1,2]. Although *G*. *polygramma* is not currently recognized as a toxin-producing species, rapid biomass accumulation can lead to water discoloration, increased organic matter, deterioration of water quality, and oxygen depletion. These high-biomass effects may cause ecological disturbances in coastal ecosystems and may also lead to physiological stress in cultured organisms or indirect impacts on aquaculture in coastal regions where aquaculture activities are intensive [3,4,5,6].

High-density blooms of *G*. *polygramma* have been reported in various coastal regions worldwide. For example, mass occurrences accompanied by fish and shellfish mortality have been documented in Uwajima Bay, Japan [3], and high-density blooms have also been reported in the Gulf of California, Mexico [7], Ambon Bay, Indonesia [8], and the southeastern Arabian Sea [4,5,9]. In Korea, following the first report of a *G*. *polygramma* bloom in the Yeosu coastal area [10], high-density blooms have been recorded mainly in offshore open coastal waters along the southern coast during summer [11]. To date, direct economic losses to aquaculture caused by *G*. *polygramma* have not been clearly reported in Korean coastal waters. However, considering previous reports of high-density *G*. *polygramma* blooms accompanied by fish and shellfish mortality in other regions, this species should be considered a bloom-forming dinoflagellate with potential ecological risk when favorable environmental conditions are established.

The southern coast of Korea is characterized by intensive shellfish and fish aquaculture, making it particularly vulnerable to fisheries impacts when harmful algal blooms (HABs) occur [12]. Dinoflagellate communities in this region exhibit strong seasonal variability, and dominant species and bloom patterns may differ depending on hydrographic conditions such as temperature, salinity, stratification, and the degree of coastal openness. Therefore, *G*. *polygramma* should be considered not only as a bloom-forming species but also in terms of its spatial and temporal occurrence patterns within the HAB monitoring framework along the southern coast of Korea. Such information can provide a basis for evaluating the potential for high-density bloom formation and assessing the ecological risk of this species in aquaculture-intensive coastal waters.

However, accurate species-level identification of *G*. *polygramma* in field samples using conventional light microscopy remains challenging. Detailed examination of thecal plate arrangement, thecal surface ornamentation, cingular displacement, and antapical spine morphology can distinguish *G*. *polygramma* from other *Gonyaulax* species [13,14,15]. Nevertheless, in routine microscopic monitoring of Lugol-fixed field samples, these diagnostic characters may become unclear depending on cell orientation, physiological condition, fixation and preservation processes, and low cell density. This limitation is particularly relevant within the genus *Gonyaulax*, in which several species share similar morphological characteristics, including cell shape, reticulated thecal surfaces, and antapical spines [15].

This issue is important because morphologically similar *Gonyaulax* species may differ in their ecological and toxicological significance. For example, *G*. *polygramma* and *G*. *spinifera* may appear similar under routine light microscopy, although they can be distinguished by detailed morphological characters such as the size and shape of antapical spines, thecal plate features, and surface ornamentation. In particular, *G*. *spinifera* has been reported as a yessotoxin (YTX)-producing species [16,17], indicating that accurate discrimination of morphologically similar *Gonyaulax* species is important for HAB monitoring. YTX-producing dinoflagellates have also been associated with mortality of cultured organisms in some coastal regions [18]. In contrast, *G*. *polygramma* is not currently recognized as a toxin-producing species and is mainly associated with high-biomass effects such as oxygen depletion and ecological disturbance. Therefore, species-specific and quantitative analytical methods are needed to complement conventional microscopy in coastal environments where morphologically similar *Gonyaulax* species may co-occur.

Quantitative polymerase chain reaction (qPCR) is a molecular technique that enables sensitive and specific detection and quantification of target species based on species-specific DNA sequences [19,20,21,22]. This method allows reliable detection even at low abundance levels that are difficult to identify microscopically and provides quantitative data through standard curve-based analysis [23,24]. These advantages make qPCR particularly suitable for monitoring species such as *G*. *polygramma*, which may occur at low densities or be confused with morphologically similar *Gonyaulax* species during routine microscopic observation. Therefore, species-specific quantification of *G*. *polygramma* in field samples from the southern coast of Korea is needed to evaluate its distribution and to assess the usefulness of molecular monitoring tools that can complement morphology-based identification.

In this study, we developed a species-specific TaqMan qPCR assay for the detection and quantification of *G*. *polygramma* and applied it to field samples collected from two coastal regions with contrasting environmental characteristics along the southern coast of Korea. Field samples were collected monthly from March to November during 2021–2022 in Jinhae Bay, a semi-enclosed embayment, and from June to August during 2021–2024 in the offshore-influenced Tongyeong–Yeosu–Wando coastal region. Using this approach, we aimed to investigate the spatiotemporal distribution patterns of *G*. *polygramma* and to evaluate whether the developed qPCR assay can serve as a molecular tool for species-specific detection of *G*. *polygramma* among morphologically similar *Gonyaulax* species.

## 2. Materials and Methods

### 2.1. Study Area and Seawater Sampling

**Figure 1:**
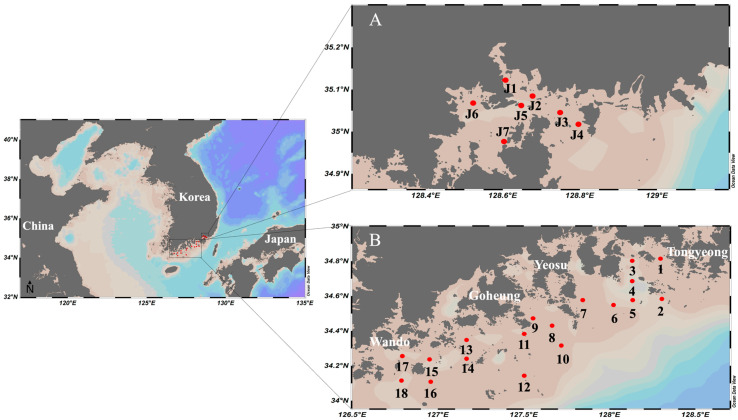
Locations of the 25 seawater sampling stations along the southern coast of Korea: (**A**) Jinhae Bay and (**B**) the Tongyeong–Yeosu–Wando coastal region. The samples were used for environmental DNA monitoring of *G. polygramma*.

This study was conducted in two coastal regions along the southern coast of Korea with different marine environmental characteristics: Jinhae Bay and the Tongyeong–Yeosu–Wando coastal area (Figure 1, Supplementary Table S1). Field surveys in both regions were carried out within the regular marine ecosystem and harmful algal bloom (HAB) monitoring programs conducted by the National Institute of Fisheries Science (NIFS). However, the survey objectives and sampling periods differed between the two regions. The Jinhae Bay survey was conducted as part of a coastal ecosystem monitoring program to investigate the seasonal occurrence of harmful algal species in a semi-enclosed embayment, whereas the Tongyeong–Yeosu–Wando survey was conducted as part of a wide-area red tide monitoring program during summer along the southern coast of Korea.

Jinhae Bay is a semi-enclosed embayment characterized by restricted water exchange with offshore waters, complex water circulation, relatively long water residence time, and strong spatial variability in water quality [25,26,27]. This region is also an important shellfish aquaculture area, and paralytic shellfish poisoning (PSP) events have been reported in association with harmful dinoflagellates, including *Alexandrium* spp. [28]. In contrast, the Tongyeong–Yeosu–Wando coastal area represents open southern coastal waters that are more directly connected to offshore waters. This region is also a major aquaculture production area where red tides are frequently reported, requiring continuous monitoring of harmful algal blooms during summer [12,29].

The two regions were not surveyed with the same sampling frequency, seasonal coverage, or year range because the surveys were designed for different monitoring purposes. In Jinhae Bay, monthly surveys were conducted from March to November during 2021–2022 to investigate the seasonal occurrence of harmful algal species, and samples were collected from seven stations. In the Tongyeong–Yeosu–Wando coastal area, surveys were conducted from June to August during 2021–2024 as part of summer red tide monitoring, and samples were collected from 18 stations. Thus, a total of 25 stations were included in this study. These datasets were therefore used to evaluate the occurrence and distribution of *G*. *polygramma* within each monitoring framework, rather than to make a seasonally matched comparison between the two regions.

All seawater samples were collected at approximately 1 m depth using a 5 L Niskin sampler. Surface samples were used because this study was based on samples collected through regular HAB monitoring programs. In such monitoring programs, repeated surveys must be conducted across multiple stations over broad coastal areas; therefore, surface sampling is suitable for maintaining consistency in spatial comparisons among stations. In addition, surface water discoloration and surface cell abundance are practical field indicators commonly used in wide-area red tide monitoring.

For molecular analysis, seawater samples were filtered immediately after collection using glass fiber filters. The filters were frozen on board, transported to the laboratory, and stored at −60 °C until DNA extraction. For microscopic analysis, separate seawater samples were collected and fixed with Lugol’s iodine solution before analysis.

Water temperature and salinity were measured in situ using a CTD profiler (Sea-Bird Scientific, Bellevue, WA, USA). Chlorophyll-a concentration was measured after filtration and extraction in 90% acetone for 24 h in the dark using a fluorometer (Turner Designs 10-AU Fluorometer, Sunnyvale, CA, USA). Dissolved inorganic nutrients, including ammonium, nitrite, nitrate, phosphate, and silicate, were analyzed using an automated nutrient analyzer (QuAAtro39, SEAL Analytical GmbH, Norderstedt, Germany).

### 2.2. Microalgal Cultures and DNA Extraction

*G*. *polygramma* (LIMS-PS-3347) was obtained from the Library of Marine Samples (LIMS) at the Korea Institute of Ocean Science and Technology (KIOST). Cultures were maintained in sterile 250 mL flasks containing 25 mL of f/2 medium (Thermo Fisher Scientific, Waltham, MA, USA). The culture conditions were set at 20 °C and salinity 30 under white fluorescent light at 100 μmol photons $m^{-2}\;s^{-1}$ with a 12 h light:12 h dark cycle.

Genomic DNA from cultured cells was extracted using the AccuPrep^®^ Genomic DNA Extraction Kit (Bioneer, Daejeon, Republic of Korea) according to the manufacturer’s protocol. DNA concentration and purity were measured using a NanoDrop™ One spectrophotometer (Thermo Fisher Scientific Inc., Waltham, MA, USA). The extracted DNA was diluted to a final concentration of 10 ng ${\mu L}^{-1}$ with TE buffer and stored at −20 °C until use.

Environmental DNA (eDNA) was extracted from filters obtained from field samples. Before DNA extraction, cells retained on the filters were mechanically disrupted using an Omni Bead Ruptor 12 Bead Mill Homogenizer (OMNI International, Kennesaw, GA, USA). Each filter was placed in a screw-cap bead-beating tube containing one 5 mm stainless steel bead (Innogentec, Gimpo, Republic of Korea) and homogenized at 4.0 m $s^{-1}$ for 1 min. DNA was then extracted using the DNeasy Blood & Tissue Kit (Qiagen, Hilden, Germany) according to the manufacturer’s animal tissue protocol. The extracted eDNA was stored at −60 °C until qPCR analysis.

### 2.3. 28S rDNA Sequencing and qPCR Marker Design

To develop a species-specific qPCR assay for *G*. *polygramma*, the nuclear 28S rDNA region was obtained from the cultured strain (LIMS-PS-3347). Genomic DNA extracted from the culture was used as a template, and the LSU rDNA D1–D3 region was amplified using the universal primer pair D1R/D3B [30,31]. This primer pair was used only for PCR amplification and sequencing of the target 28S rDNA region. The expected amplicon size of the LSU rDNA D1–D3 region varies among taxa, but is generally within the range of approximately 1.0–1.4 kb.

PCR amplification was performed using AccuPower^®^ PCR PreMix (Bioneer, Daejeon, Republic of Korea) on a ProFlex PCR System (Thermo Fisher Scientific Inc., Waltham, MA, USA). PCR products were verified by agarose gel electrophoresis and purified using the AccuPrep^®^ PCR Purification Kit (Bioneer, Daejeon, Republic of Korea). Sequencing was conducted using the BigDye™ Terminator v3.1 Cycle Sequencing Kit (Thermo Fisher Scientific Inc., Waltham, MA, USA), and the products were analyzed on an ABI 3730xl DNA Analyzer (Thermo Fisher Scientific Inc., Waltham, MA, USA). Both forward and reverse primers used for PCR amplification were also used for sequencing.

The obtained sequences were edited and assembled using BioEdit version 7.2.5. Low-quality regions at both ends were trimmed, and forward and reverse sequences were aligned to generate a consensus sequence. The 28S rDNA sequence of *G*. *polygramma* was aligned with sequences of closely related dinoflagellates retrieved from the GenBank database using the ClustalW algorithm in BioEdit. Species-specific variable regions were identified from the aligned sequence matrix and selected as candidate target regions for TaqMan qPCR primer and probe design.

Species-specific qPCR primers and a TaqMan probe were designed using Primer3Web version 4.1.0. Primer specificity was evaluated using NCBI Primer-BLAST (https://www.ncbi.nlm.nih.gov/tools/primer-blast/, accessed on 17 May 2024), while the GC content and melting temperature (Tm) of the primers and probe were assessed using PCR Primer Stats in Sequence Manipulation Suite version 2 (SMS2; https://www.bioinformatics.org/sms2/pcr_primer_stats.html, accessed on 30 May 2024). Candidate qPCR primers were selected to have lengths of 17–21 bp, GC contents of 38–59%, and melting temperatures (Tm) of 60–63 °C. The TaqMan probe was designed to be 24 bp in length, with a GC content below 46% and a Tm approximately 3–6 °C higher than that of the primers. These design criteria were applied to the newly developed *G*. *polygramma*-specific qPCR primer–probe candidates, not to the universal D1R/D3B primers used for 28S rDNA sequencing. Potential formation of self-dimers, heterodimers, and hairpins was evaluated to minimize non-specific amplification. The final primers and probe were synthesized (Bioneer, Daejeon, Republic of Korea).

### 2.4. qPCR Conditions and Selection of Candidate Markers

qPCR assays were performed using a QuantStudio™ 5 Real-Time PCR System (Thermo Fisher Scientific Inc., Waltham, MA, USA). Each reaction was prepared in a total volume of 20 μL containing 10 μL of 2× GoTaq^®^ Probe qPCR Master Mix (Promega, Madison, WI, USA), 2 μL of template DNA, 1 μL of forward primer, 1 μL of reverse primer, 0.5 μL of TaqMan probe, 0.2 μL of CXR reference dye, and 5.3 μL of nuclease-free water. The stock concentrations of the primers and probe were 5 pmol ${\mu L}^{-1}$, resulting in final concentrations of 250 nM for each primer and 125 nM for the TaqMan probe (Supplementary Table S5). Cultured genomic DNA or environmental DNA was used as the template, and all reactions were conducted in triplicate.

The thermal cycling conditions consisted of an initial denaturation at 95 °C for 2 min, followed by 40 cycles of 95 °C for 15 s and 60 °C for 45 s. Fluorescence signals were measured in the FAM channel, and Ct values and amplification curves were analyzed using QuantStudio Design and Analysis Software version 1.5.2 (Thermo Fisher Scientific Inc., Waltham, MA, USA). Each qPCR run included a positive control and a no-template control (NTC) to assess amplification performance, contamination, and non-specific reactions.

A total of six candidate primer–probe sets were evaluated under identical qPCR conditions. Initial screening was based on the presence of clear amplification curves in the positive control and the absence of amplification in the NTC. The final primer–probe set was selected based on species specificity inferred from sequence alignment, stability of amplification curves, and reproducibility of Ct values.

### 2.5. Specificity Testing of the qPCR Assay

The specificity of the developed qPCR assay was evaluated using genomic DNA from *G*. *polygramma* and non-target microalgal cultures (Supplementary Table S2). The non-target species were selected because they are known or likely to co-occur with *G*. *polygramma* in the study area. Genomic DNA extracted from each culture was adjusted to a concentration of 5 ng ${\mu L}^{-1}$, and 2 μL was used as the template in each qPCR reaction, corresponding to 10 ng ${reaction}^{-1}$. All DNA samples were analyzed under the same qPCR conditions described in Section 2.4.

Specificity was evaluated by confirming amplification of *G. polygramma* and the absence of amplification in all non-target species and the no-template control (NTC). The assay was considered specific to *G*. *polygramma* when a clear amplification signal was obtained only from the target species, with no amplification detected in any non-target species or the NTC.

### 2.6. Standard Curve Construction and Sensitivity Assessment

To evaluate the quantitative performance of the developed qPCR assay, cultured cells of *G*. *polygramma* and plasmid DNA were used as standard materials. Culture-based standards were prepared by serial tenfold dilutions of cell suspensions with known cell concentrations. Plasmid standards were prepared using a synthesized 28S rDNA fragment containing the primer–probe binding region, which was constructed as plasmid DNA (Bioneer, Daejeon, Republic of Korea). The plasmid standards were then diluted in a tenfold series. Each dilution was analyzed in triplicate under the qPCR conditions described in Section 2.4.

The culture-based standard curve was generated by plotting Ct values against log_10_-transformed cell concentrations. The plasmid-based standard curve was generated by plotting Ct values against log_10_-transformed copy numbers. For each standard curve, the slope, coefficient of determination ($R^{2}$), and amplification efficiency were calculated to evaluate assay performance. The plasmid-based standard curve was used to estimate 28S rDNA copy numbers in environmental samples, whereas the culture-based standard curve was used to evaluate the relationship between cell concentration and qPCR signal. In addition, the 28S rDNA copy number per cell of *G*. *polygramma* was estimated by comparing the two standard curves. The results from environmental samples were presented separately as copy number and estimated cell abundance.

The limit of detection (LOD) was defined as the lowest concentration at which stable amplification was observed across replicate reactions. The limit of quantification (LOQ) was defined as the lowest concentration at which reproducible quantification was obtained. Analytical reproducibility was evaluated using the mean, standard deviation (SD), and coefficient of variation (CV) of Ct values across replicate reactions.

### 2.7. Quantification of Environmental Samples

Environmental DNA extracted from field samples was analyzed using the selected *G*. *polygramma*-specific primer–probe set under the qPCR conditions described in Section 2.4. All samples were analyzed in triplicate, and a no-template control (NTC) was included in each qPCR run to monitor possible contamination and non-specific amplification. A sample was considered positive only when a clear amplification curve was observed and the Ct value fell within the valid range of the standard curve.

Quantification of environmental samples was based on copy numbers per reaction derived from the plasmid-based standard curve. These values were converted to concentrations in the original seawater samples by accounting for DNA extraction volume, template DNA volume used in the qPCR reaction, and filtered seawater volume. Final copy numbers were expressed as ${copies\;mL}^{-1}$.

In addition, copy numbers were converted to estimated ${cells\;mL}^{-1}$ using the 28S rDNA copy number per cell estimated from the comparison between the plasmid-based and culture-based standard curves. Therefore, the results from environmental samples were presented separately as ${copies\;mL}^{-1}$ and estimated ${cells\;mL}^{-1}$.

(1) $$Copy\;number\left(\frac{copies}{µL}\right)=\frac{DNA\;concentration\left(\frac{ng}{µL}\right)\times 6.022\times 10^{23}}{DNA\;length\left(bp\right)\times 660\times 10^{9}}$$

### 2.8. Microscopic Observation and Cell Enumeration

Samples for phytoplankton analysis were fixed immediately after collection with Lugol’s iodine solution at a final concentration of 1%. Fixed samples were stored in the dark and transported to the laboratory for analysis. For microscopic observation, 1 L of seawater was concentrated to a final volume of 50 mL. The concentrated samples were thoroughly mixed and transferred to a 1 mL counting chamber. The entire chamber was examined using a light microscope for identification and enumeration of phytoplankton.

*G*. *polygramma* was identified based on morphological characteristics described in previous taxonomic studies, including cell size and shape, thecal plate arrangement, thecal surface ornamentation, cingular position, and antapical spine morphology [13,14,15]. Cell abundances were calculated by considering the original sample volume, final concentrated volume, and counting volume, and were expressed as ${cells\;mL}^{-1}$.

The practical detection limit of microscopic enumeration in this study was estimated based on the concentration of 1 L of seawater to a final volume of 50 mL and the examination of the entire 1 mL counting chamber. Under these conditions, the detection of one cell in 1 mL of concentrated sample corresponds to 0.05 ${cells\;mL}^{-1}$ in the original seawater sample. Microscopic data were used together with qPCR results to interpret the occurrence and distribution patterns of *G*. *polygramma.*

## 3. Results

### 3.1. Validation of G. polygramma-Specific Primers and Probe

Species-specific qPCR primer–probe candidate sets targeting the 28S rDNA region of *G*. *polygramma* (LIMS-PS-3347) were designed and evaluated (Supplementary Figures S1–S3). A total of six candidate primer–probe sets were compared under the same qPCR conditions. Amplification of the positive control was observed in all candidate sets; however, Set 5 showed amplification in the no-template control (NTC) and was therefore excluded from further analysis (Supplementary Figure S4). Among the remaining candidate sets, Set 6 showed stable amplification curves for the positive control, with no amplification in the NTC. Therefore, Set 6 was selected as the final *G*. *polygramma* specific primer–probe set (Table 1).

The specificity of the selected Set 6 was further evaluated using genomic DNA from *G*. *polygramma* and 23 non-target microalgal species, including three toxic phytoplankton species and twenty non-toxic microalgal species. Amplification was detected only in *G*. *polygramma*, whereas no amplification was observed in any non-target species or in the NTC (Supplementary Figure S5, Table S2). These results indicate that the selected primer–probe set has high species specificity for the detection of *G*. *polygramma*.

### 3.2. Standard Curve Construction and Assay Performance

**Figure 2:**
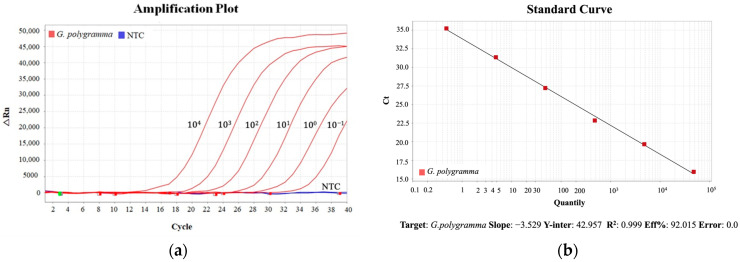
qPCR amplification plots and standard curve for *G*. *polygramma* based on culture-derived DNA. (**a**) Amplification plots showing serial dilutions of target DNA, with no amplification observed in the negative control (NTC). The green and red boxes indicate software-defined baseline regions for different fluorescence channels. (**b**) Standard curve constructed from Ct values versus log-transformed cell concentrations, showing high linearity.

To evaluate the quantitative performance and sensitivity of the developed qPCR assay, both culture-based and plasmid-based standard curves were constructed. The culture-based standard curve was generated using tenfold serial dilutions ranging from 4.8 × $10^{4}$ to 4.8 × $10^{-2}\;{cells\;reaction}^{-1}$ (Figure 2a). Clear increases in Ct values corresponding to decreasing cell concentrations were observed over the range of 4.8 × $10^{4}$–4.8 × $10^{-1}\;{cells\;reaction}^{-1}$, whereas no amplification was detected in the NTC. The regression equation for the culture-based standard curve was y = −3.529x + 42.957, with a coefficient of determination of R^2^ = 0.999 (Figure 2b). The calculated amplification efficiency was 92.01%. Replicate analyses showed Ct standard deviations ranging from 0.07 to 0.14. Amplification was consistently detected even at 4.8 × $10^{-1}$ ${cells\;reaction}^{-1}$, with a mean Ct value of 35.25 ± 0.20. In contrast, amplification at 4.8 × $10^{-2}\;{cells\;reaction}^{-1}$ was inconsistent and classified as inconclusive or undetermined.

**Figure 3:**
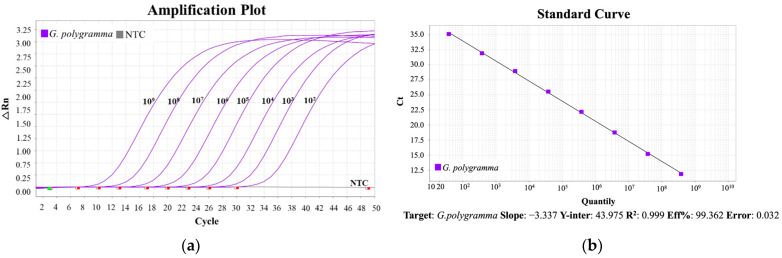
qPCR amplification plots and standard curve for *G*. *polygramma* based on plasmid DNA. (**a**) Amplification plots showing serial dilutions of plasmid DNA, with no amplification observed in the negative control (NTC). The green and red boxes indicate software-defined baseline regions for different fluorescence channels. (**b**) Standard curve constructed from Ct values versus log-transformed copy numbers, showing high linearity.

A plasmid-based standard curve was subsequently generated using synthesized 28S rDNA fragments containing the target amplification region (Figure 3a). The regression equation between Ct values and log_10_-transformed copy numbers was y = −3.337x + 43.975, with an R^2^ value of 0.999 and an amplification efficiency of 99.36% (Figure 3b). Amplification was detected down to 10 ${copies\;reaction}^{-1}$. Based on these analyses, the limit of detection (LOD) was determined to be ${copies\;reaction}^{-1}$, while the limit of quantification (LOQ) was estimated as 10^2^ copies reaction^−1^. The analytical LOD of the qPCR assay corresponded to an estimated cell-equivalent detection level of approximately 0.0083 ${cells\;mL}^{-1}$ under the analytical conditions used in this study. This value was lower than the practical detection limit of microscopic enumeration, which was estimated as 0.05 ${cells\;mL}^{-1}$ based on the concentration and counting procedure described in Section 2.8.

Comparison of the culture-based and plasmid-based standard curves indicated that the 28S rDNA copy number of *G*. *polygramma* was approximately 2.4 × $10^{2}\;{copies\;mL}^{-1}$. This conversion factor was subsequently used to estimate cell abundance in environmental samples.

### 3.3. qPCR Detection of G. polygramma in Jinhae Bay

**Figure 4:**
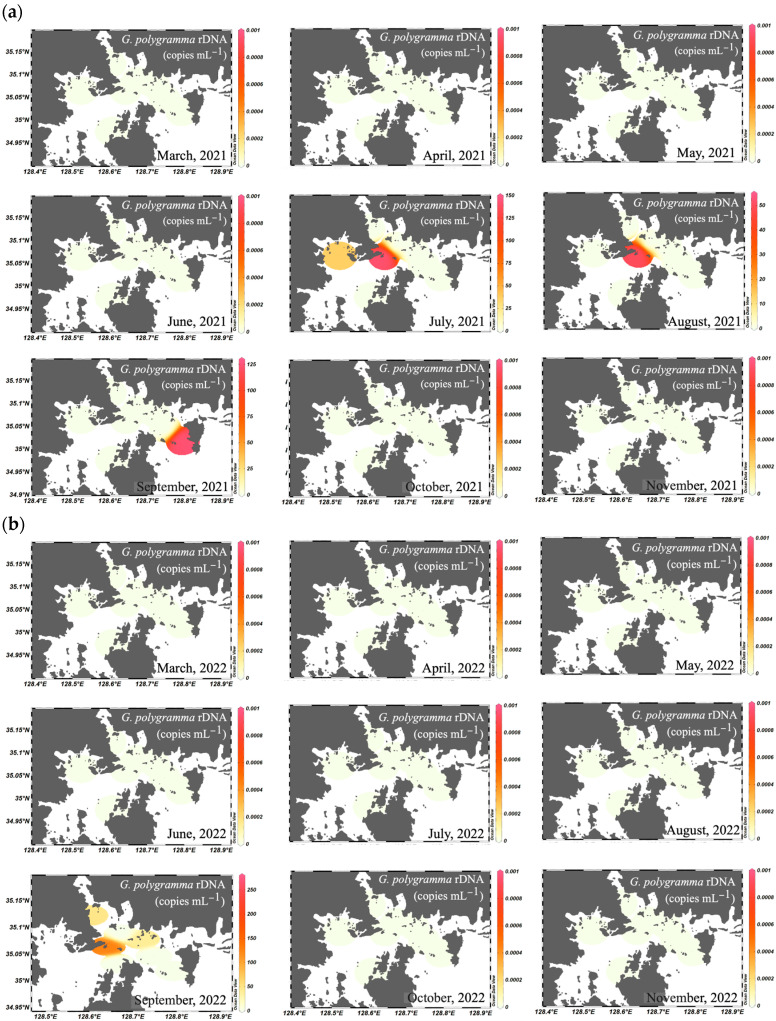
Monthly distribution of *G*. *polygramma* quantified by qPCR (${copies\;mL}^{-1}$) in Jinhae Bay during the monitoring periods from March to November in 2021 and 2022: (**a**) 2021 and (**b**) 2022.

To investigate the occurrence of *G*. *polygramma* in Jinhae Bay, surface seawater samples collected between March and November during 2021–2022 were analyzed using qPCR (Table 2; Figure 4). During the study period, *G*. *polygramma* was rarely detected, and positive signals were limited primarily to several summer and early autumn samples.

In 2021, no clear qPCR amplification was observed from March to June or from October to November, whereas localized detections were identified at several stations during July–September (Figure 4). Quantified abundances ranged from 45 to 142 ${copies\;mL}^{-1}$ in July, 50.3 ${copies\;mL}^{-1}$ in August, and 123 ${copies\;mL}^{-1}$ in September (Table 2). These values corresponded to estimated abundances of approximately 0.2–0.59, 0.2, and 0.51 ${cells\;mL}^{-1}$, respectively. In 2022, *G*. *polygramma* was detected only in September, with abundances ranging from 81 to 249 ${copies\;mL}^{-1}$, corresponding to estimated cell concentrations of 0.34–1.04 ${cells\;mL}^{-1}$. Overall, qPCR-derived abundances in Jinhae Bay remained consistently low throughout both sampling years.

Microscopic observations of the same samples identified *G*. *polygramma*-like cells at concentrations ranging from 0 to 7.3 ${cells\;mL}^{-1}$ in 2021 and from 0 to 6.8 ${cells\;mL}^{-1}$ in 2022 (Table 2). Notably, cells morphologically identified as *G*. *polygramma* were occasionally observed during spring months, although qPCR analysis of the same samples yielded no amplification signal. Conversely, even during periods when qPCR amplification was detected, microscopic counts remained low. Environmental conditions during periods of qPCR detection generally corresponded to summer and early autumn conditions. In 2021, detection occurred at water temperatures of approximately 22–26 °C and salinities of 28–32. In September 2022, detection occurred at a temperature of approximately 23 °C and a salinity of 29.3.

### 3.4. qPCR Detection of G. polygramma in the Tongyeong–Yeosu–Wando Coastal Region

**Figure 5:**
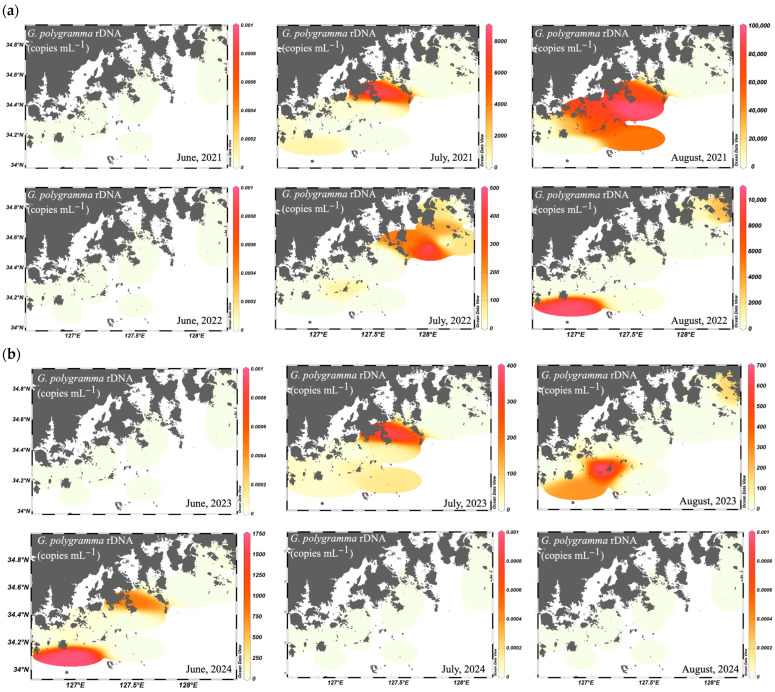
Monthly distribution of *G*. *polygramma* quantified by qPCR (${copies\;mL}^{-1}$) in the Tongyeong–Yeosu–Wando coastal region during the summer monitoring periods from June to August in 2021–2024: (**a**) 2021–2022 and (**b**) 2023–2024.

The summer occurrence patterns of *G*. *polygramma* in the Tongyeong–Yeosu–Wando coastal region were investigated using surface seawater samples collected between June and August during 2021–2024 (Figure 5; Table 3). qPCR analysis revealed clear interannual and monthly variability in abundance, with higher values than those observed in Jinhae Bay.

In 2021, *G. polygramma* was not detected by either qPCR or microscopy in June, but was detected at several stations in July and increased markedly in August. qPCR-derived abundances ranged from 144 to 8016 ${copies\;mL}^{-1}$ in July, corresponding to estimated abundances of 0.6–33.4 ${cells\;mL}^{-1}$. In August, abundances increased substantially to 19,896–97,176 ${copies\;mL}^{-1}$, corresponding to estimated concentrations of 82.9–404.9 ${cells\;mL}^{-1}$, which were the highest values observed during the study period. Microscopic observations also showed relatively high abundances in 2021, ranging from 0 to 24.6 ${cells\;mL}^{-1}$ in July and from 21.6 to 298 ${cells\;mL}^{-1}$ in August.

In 2022, *G*. *polygramma* was detected continuously from June through August. Quantified abundances ranged from 72 to 3816 ${copies\;mL}^{-1}$ in June, 72 to 480 ${copies\;mL}^{-1}$ in July, and 216 to 10,368 ${copies\;mL}^{-1}$ in August. Estimated cell abundances were 0.3–15.9, 0.3–2.0, and 0.9–43.2 ${cells\;mL}^{-1}$, respectively. Microscopic abundances ranged from 0 to 10 ${cells\;mL}^{-1}$ in June, 0 to 1 ${cells\;mL}^{-1}$ in July, and 0 to 65 ${cells\;mL}^{-1}$ in August.

In 2023, *G*. *polygramma* was not detected by qPCR in June, while low qPCR abundances were observed in July and August. qPCR-derived abundances ranged from 0 to 350 ${copies\;mL}^{-1}$ in July and 144 to 696 ${copies\;mL}^{-1}$ in August, corresponding to estimated abundances of 0–1.5 and 0.6–2.9 ${cells\;mL}^{-1}$, respectively. Microscopic abundances remained low, ranging from 0 to 0.2 ${cells\;mL}^{-1}$ in June, 0 to 0.8 ${cells\;mL}^{-1}$ in July, and 0.4 to 2 ${cells\;mL}^{-1}$ in August.

In 2024, qPCR detection occurred only in June, with abundances ranging from 192 to 1692 ${copies\;mL}^{-1}$, corresponding to estimated abundances of 0.8–7.1 ${cells\;mL}^{-1}$. No qPCR amplification was detected in July or August. Microscopic observations showed 0.1–1 ${cells\;mL}^{-1}$ in June and 1 ${cells\;mL}^{-1}$ in July, whereas no cells were detected microscopically in August.

Samples with relatively high qPCR abundances were observed at water temperatures of 21–28 °C, salinities of 30–33, and station depths of 20–40 m. Particularly high abundances, including values exceeding $10^{4}$–$10^{5}$ ${copies\;mL}^{-1}$, were observed at several stations during August 2021. Overall, the qPCR results showed that *G*. *polygramma* detection in the Tongyeong–Yeosu–Wando coastal region was concentrated in specific years, months, and stations rather than being evenly distributed throughout the study period.

## 4. Discussion

### 4.1. Analytical Reliability and Interpretation of the qPCR Assay

In this study, we developed a species-specific TaqMan qPCR assay for the detection and quantification of *Gonyaulax polygramma* and applied the assay to environmental samples collected from Korean coastal waters. Conventional microscopic observation remains the fundamental method for phytoplankton monitoring because it allows direct examination of cell morphology and community composition. However, detailed diagnostic characteristics can become unclear in preserved field samples depending on cell orientation, preservation condition, and low cell density. This limitation is particularly relevant within the genus *Gonyaulax*, in which several species share similar morphological characteristics, including cell shape, reticulated thecal surfaces, cingular displacement, and antapical spines. Therefore, reliable species-level identification based only on routine light microscopy can be difficult in field samples.

The 28S rDNA region was selected as the molecular target because it contains both conserved and variable domains suitable for species-specific primer and probe design. Previous molecular phylogenetic studies based on the LSU rDNA D1–D3 region have been conducted for Korean *G*. *polygramma* isolates, supporting the molecular discrimination of this species [14]. In the present study, the developed primer–probe set amplified only *G*. *polygramma,* whereas no amplification was observed in the tested non-target microalgae or the NTC. In addition, both culture-based and plasmid-based standard curves showed high linearity and stable amplification efficiency. These results indicate that the developed assay has sufficient analytical reliability for confirming the presence of *G*. *polygramma* in field samples and for comparing relative differences in detection intensity among samples.

However, qPCR-derived abundance estimates require careful interpretation, as variation in rDNA copy number between cultured standards and field populations can bias estimates of cell abundance [32,33].

In this study, the plasmid-based standard curve was used to quantify 28S rDNA copy numbers in environmental samples because it provides a defined molecular standard for target copy estimation. The culture-based standard curve was used to evaluate the relationship between qPCR signal and cell concentration and to estimate an approximate copy number per cell. The slopes of the plasmid-based and culture-based standard curves were not identical, which may reflect differences in template type and preparation process between purified plasmid DNA and DNA extracted from cultured cells. Therefore, environmental results were presented separately as ${copies\;mL}^{-1}$ and estimated ${cells\;mL}^{-1}$ to avoid overinterpretation of qPCR-derived cell abundance. The estimated ${cells\;mL}^{-1}$ values should be interpreted primarily as approximate cell-equivalent values or relative indicators of detection intensity and spatial–temporal distribution, rather than as exact absolute cell concentrations. Nevertheless, the qPCR assay showed a lower analytical detection limit than microscopy and improved species-specific detection reliability compared with morphology-based observation alone.

### 4.2. Regional Differences in the Occurrence of G. polygramma Along the Southern Coast of Korea

Application of the qPCR assay to field samples revealed clear regional differences in the occurrence patterns of *G*. *polygramma*. In Jinhae Bay, the species was rarely detected or remained at low abundance levels, whereas relatively higher qPCR abundances were observed at some stations and years in the Tongyeong–Yeosu–Wando coastal region. These results indicate that *G*. *polygramma* is not uniformly distributed throughout the southern coast of Korea, but may show localized occurrence patterns depending on season and region.

The contrasting distribution patterns between the two regions may be associated with hydrographic characteristics commonly observed in offshore-influenced coastal waters during summer. Jinhae Bay is a semi-enclosed embayment characterized by restricted water exchange with offshore waters, relatively shallow depth, and long water residence time [25]. In contrast, the Tongyeong–Yeosu–Wando coastal region represents more open coastal waters connected to offshore waters, where thermal stratification can develop during summer due to surface heating, freshwater input, and offshore water influence. Therefore, the more prominent detection of *G*. *polygramma* in the Tongyeong–Yeosu–Wando coastal region than in Jinhae Bay is likely not attributable to a single environmental factor, but rather to the combined effects of seasonal water-column structure and local hydrographic variability in offshore-influenced coastal waters.

This interpretation is generally consistent with previous reports on the bloom ecology of *G*. *polygramma*. Along the southern coast of Korea, a large-scale bloom of *G*. *polygramma* was reported during the summer of 2009, and bloom formation and maintenance were suggested to be associated with stratification, weak wind conditions, high solar radiation, and nutrient conditions [11]. In other coastal regions, *G*. *polygramma* has also been reported as a species associated with water discoloration and high-density blooms [5,6,8,9,34]. In the southeastern Arabian Sea, subsurface blooms of this species were observed under stable water-column conditions, shallow mixed layers, sufficient nutrients, and high chlorophyll-a concentrations [9]. These previous studies support the interpretation that *G*. *polygramma* may show localized high-density occurrence when suitable hydrographic and environmental conditions are established, although the specific conditions may differ among coastal regions.

The ecological traits of *G*. *polygramma* may also help explain its distribution pattern. Dinoflagellates can use their motility to exploit vertical gradients of light, nutrients, and prey organisms in stratified water columns. *G*. *polygramma* is also known to have mixotrophic nutritional capability [1,2], and this ecological flexibility may contribute to its persistence and localized occurrence under variable nutrient conditions. However, the present study did not directly measure feeding activity, vertical migration, prey availability, or depth-resolved distribution. Therefore, the relatively prominent occurrence observed in the Tongyeong–Yeosu–Wando coastal region can be interpreted as an ecological association with offshore-influenced and seasonally structured coastal waters, but the mechanisms underlying this distribution should be further examined through additional studies.

### 4.3. Discrepancy Between Microscopy and qPCR and the Significance of Species Identification Within the Genus Gonyaulax

In some samples, microscopic observations and qPCR results did not correspond. In Jinhae Bay, cells morphologically identified as *G*. *polygramma* were occasionally observed microscopically, whereas qPCR detection was negative. In the Tongyeong–Yeosu–Wando coastal region, microscopic cell counts were also not always consistent with qPCR-derived abundances. These discrepancies should not be interpreted simply as errors in either method, but rather as reflecting the different characteristics and limitations of microscopy and qPCR. Microscopy allows direct observation of cell morphology and community composition; however, diagnostic features may be difficult to resolve in preserved field samples, particularly when cell abundance is low or morphologically similar species co-occur. In contrast, qPCR enables more sensitive and species-specific detection based on target DNA sequences, but it does not provide information on cell morphology or community structure. Therefore, microscopy and qPCR should be regarded as complementary approaches: microscopy remains essential for morphological observation and community-level assessment, whereas qPCR improves the sensitivity and reliability of species-specific confirmation of *G*. *polygramma*.

This issue is particularly important for species-level identification within the genus *Gonyaulax*. Detailed examination of thecal plate arrangement, thecal surface ornamentation, and antapical spine morphology can distinguish *G*. *polygramma* from other *Gonyaulax* species [13,14]. However, these characters are not always clearly observed during routine light microscopy. For example, *G*. *polygramma* and *G*. *spinifera* may appear morphologically similar, although they can be distinguished by detailed characters such as the size and shape of antapical spines, thecal plate features, and surface ornamentation [15].

Accurate species discrimination is also important for ecological and toxicological interpretation. *G*. *polygramma* has not been reported as a toxin-producing species, but high-biomass blooms of this species have been associated with water discoloration, oxygen depletion, and associated ecological disturbance [3,6]. In contrast, some *Gonyaulax* species, including *G*. *spinifera*, have been reported as yessotoxin (YTX)-producing species [16,17]. YTX-producing dinoflagellates have also been associated with mortality of cultured organisms in some coastal regions [18]. Therefore, accurate species-level identification is important for interpreting HAB monitoring data in coastal environments where morphologically similar *Gonyaulax* species may co-occur.

The qPCR assay developed in this study can be used as a complementary tool to reduce uncertainty in morphology-based identification. The assay is not intended to replace microscopic observation, but rather to independently confirm the presence of *G*. *polygramma* in field samples. However, the specificity test in this study did not include all potentially co-occurring *Gonyaulax* species. In particular, some closely related species such as *G*. *spinifera* were not experimentally tested. Therefore, the possibility of cross-reactivity with untested *Gonyaulax* species cannot be completely excluded. Further specificity testing using a broader range of *Gonyaulax* species, together with sequence-based confirmation of field populations, will be necessary. In addition, some microscopy-positive but qPCR-negative samples may have contained morphologically similar *Gonyaulax* species that could not be reliably distinguished by routine light microscopy.

### 4.4. Study Limitations and Future Perspectives

This study developed a species-specific qPCR assay for *G*. *polygramma* and applied it to field samples from the southern coast of Korea, revealing regional differences in the occurrence of this species. However, several limitations should be considered. First, qPCR-based estimated cell abundance can be affected by variation in rDNA copy number; therefore, it should be interpreted as a relative distribution indicator rather than as an exact absolute cell concentration. Second, this study was based mainly on surface samples collected through regular HAB monitoring programs, and therefore did not fully evaluate the vertical distribution of *G*. *polygramma*. Third, stratification intensity, light conditions, prey availability, and nutrient dynamics were not directly examined in this study.

Future studies should combine species-specific qPCR analysis with depth-resolved sampling and measurements of water-column stratification, light conditions, nutrient variability, and prey distribution. Comparative analyses including morphologically similar *Gonyaulax* species, particularly potential YTX-producing species such as *G*. *spinifera*, are also needed. Such approaches will help clarify the local occurrence conditions of *G*. *polygramma* along the southern coast of Korea and improve the ecological interpretation and monitoring accuracy of *Gonyaulax* species in coastal HAB monitoring programs.

## 5. Conclusions

Overall, the present study developed a species-specific TaqMan qPCR assay for the detection and quantification of *G*. *polygramma* in Korean coastal waters. The assay showed high specificity, linearity, and amplification efficiency, and successfully detected *G. polygramma* in field samples. Field application revealed that *G*. *polygramma* was rarely detected in Jinhae Bay, whereas it was detected more frequently and at higher abundances in the Tongyeong–Yeosu–Wando coastal region during summer. These results suggest that *G*. *polygramma* may occur locally in offshore-influenced coastal waters rather than being uniformly distributed along the southern coast of Korea. The developed qPCR assay provides a lower detection limit than microscopy and improves the reliability of species-specific confirmation of *G*. *polygramma*, particularly when morphologically similar *Gonyaulax* species may co-occur in field samples. Further studies, including depth-resolved sampling and additional specificity testing with related *Gonyaulax* species, are needed to better understand its ecological occurrence patterns.

## Figures and Tables

**Table 1:** Sequence information for the *G*. *polygramma* (LIMS-PS-3347)-specific primer set.

Target Species	Primer/Probe	Sequence (5′ → 3′)
*G*. *polygramma* (LIMS-PS-3347)	Forward	CCAGACATGCATAAGTGACA
	Reverse	ATTACACCCCAAATGATGACT
	Probe	FAM-TATGCAGCTTTGTCTTTGTCGCTG-BHQ1

**Table 2:** qPCR and microscopic observation results for *G*. *polygramma* detected in Jinhae Bay from March to November during 2021–2022.

Year	Month	qPCR ${copies\;mL}^{-1}$	Estimated Cell Density ${cells\;mL}^{-1}$	Microscopy ${cells\;mL}^{-1}$
2021	3	ND	ND	0.9–7.3
	4	ND	ND	0–5.2
	5	ND	ND	0–2.4
	6	ND	ND	0–0.1
	7	45–142	0.2–0.6	0–4.5
	8	50.3	0.2	0–2.2
	9	123	0.51	0–0.1
	10	ND	ND	0–0.1
	11	ND	ND	0
2022	3	ND	ND	0–6.8
	4	ND	ND	0–5.2
	5	ND	ND	0–2.2
	6	ND	ND	0–4.5
	7	ND	ND	0–4.5
	8	ND	ND	0–0.4
	9	81–249	0.3–1.0	0–2
	10	ND	ND	0–0.1
	11	ND	ND	0–0.2

ND—Not detected.

**Table 3:** qPCR and microscopic observation results for *G*. *polygramma* detected in the Tongyeong–Yeosu–Wando coastal region from June to August during 2021–2024.

Year	Month	qPCR ${copies\;mL}^{-1}$	Estimated Cell Density ${cells\;mL}^{-1}$	Microscopy ${cells\;mL}^{-1}$
2021	June	ND	ND	ND
	July	144–8016	0.6–33.4	0–24.6
	August	19,896–97,176	82.9–404.9	21.6–298
2022	June	72–3816	0.3–15.9	0.6–10
	July	72–480	0.3–2	0.2–1
	August	216–10,368	0.9–43.2	0.2–65
2023	June	ND	ND	0.1–0.2
	July	0–350	0–1.5	0.1–0.8
	August	144–696	0.6–2.9	0.4–2
2024	June	192–1692	0.8–7.1	0.1–1.1
	July	ND	ND	1
	August	ND	ND	ND

ND—Not detected.

## Data Availability

The datasets generated and/or analyzed in the current study are available from the corresponding author upon reasonable request.

## Supplementary Materials

The following supporting information can be downloaded at: https://www.mdpi.com/article/10.3390/biology15131048/s1, Figure S1: Absorbance spectrum showing the concentration and purity of DNA extracted from agarose gel images of the amplified PCR products; Figure S2: Absorbance spectrum showing the concentration and purity of DNA extracted from *G*. *polygramma* culture; Figure S3: DNA sequencing results of *G*. *polygramma* (LIMS-PS-3347); Figure S4: *G*. *polygramma* amplification plot showing the qPCR reactions performed using species-specific PCR primer sets and fluorescent probes (molecular marker sets) that were originally designed for the target species; Figure S5: Specificity test of the molecular marker using 23 reference species and *G*. *polygramma* strain (LIMS-PS-3347); Table S1: Sampling information and locations of the 25 seawater stations; Table S2: List of non-target phytoplankton species used for assay specificity testing; Table S3: Global occurrence patterns of *G. polygramma* and associated impacts on marine organism mortality in diverse geographical regions; Table S4: Composition of standard PCR mixture; Table S5: Composition of the qPCR mixture for the detection of *G*. *polygramma*; Table S6: PCR amplification results of *G*. *polygramma* using species-specific primer–probe sets.
