# Supplementary material for: Species-Specific qPCR Detection Reveals Offshore Distribution of Gonyaulax polygramma (Dinophyceae) in Korean Coastal Waters
Source: Biology (Basel). 2026 Jul 1;15(13):1048. doi: 10.3390/biology15131048 (PMC13360600; doi:10.3390/biology15131048)
Supplement: Supplementary file 1 [file biology-15-01048-s001.zip › biology-4349414-supplementary.pdf]

**Supplementary Figures**

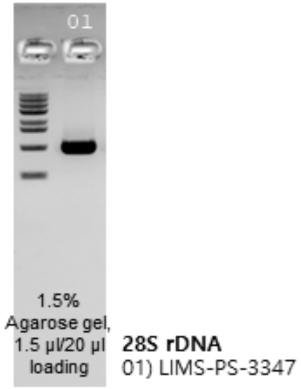


**Figure S1.** Absorbance spectrum showing the concentration and purity of DNA extracted from agarose gel images of the amplified PCR products.

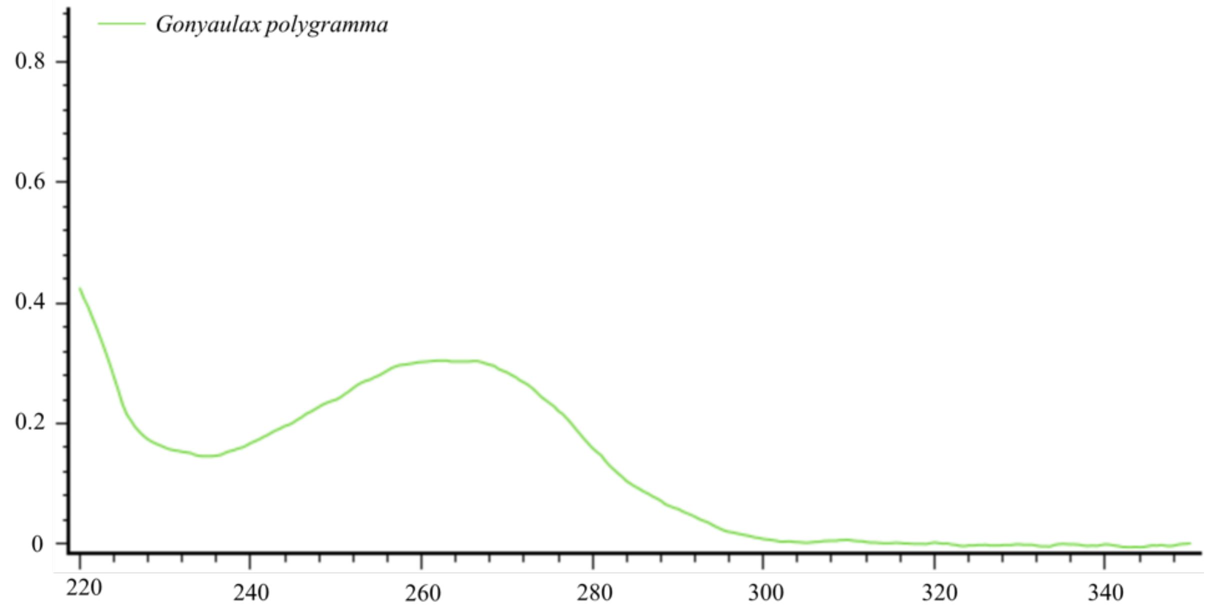


**Figure S2.** Absorbance spectrum showing the concentration and purity of DNA extracted from *G. polygramma* culture.

*Gonyaulax polygramma* (LIMS-PS-3347)

GATGGTTCGATTAGTCTTTCGCCCCTATACCCAAGTATGACGAACGATTTGCACGTCAGTACCGATGCAAGCCTCCATCAGAGTTTCCCCTGAATTT
ACTTTTCTCAAGCATAGTTCACCATCTTTCGGGTCCTAACACAAATGCTCAAGCTCAAACCTCTCACAATTGATTGGTTGGTTGGTGGTGCGCAAAT
CCCACCTGTCATTTTCATTGTGCCAACAAATCTAAATTCATAAACTCGCACATGTGTTAGACTCCTTGGTCCGTGTTTCAAGACGGGTCAAAAGAA
ATATCATCACCAAAGAACACATCAGGGAAACACGAGCAAAACATGCTCAGGCAGCCCAGATGTACAGCAATCAAACAGGATGCCAGACGAAGG
AGCAATCATTCAAAAAGTTGAAGCCCCTTGCACCATGCACATTGGCAAGACCTAAATGATGTTTGACATGCAAAGGCAGACACACCACACAGGT
GAGGAGAAGGGACACTTAACAGCAAGTGTGCGAAGTCGCCACCCCAAACACATGAAGCAATCTACCTCAACAAGATCAATTTCGTTTGCTTTCTT
TTCAACAATTTCAGGTACTTCTAACTTTCTTTTCAAAGTCCTTTTCACTTTTCCCTCATGGTACTTGTTTGCTATCAATTTCAAACCCATATTCAGCTTT
AGATGAAATTTACCACCCACTTTGCGCTCCAGTTTCAGGGAACACGATTCGTGGAAGCATGCCATACATAGCAAGCCACAAGGACACACAGGA
CTTTCACCTTCAATGCTGCTTCACCCCAAAAGCTTACCCCTGCATTGCTATTGGCATTGCATCATAAACATACAATTCAAACCTGAAAGGCTTGATT
TTCAGCATGAGCTTTTCCTTGTTCATTCGCCATTACTACAAGAATCCTGTTCAGTGTGTTGTCCTCCGCTTACTTA

**Figure S3.** DNA sequencing results of *G. polygramma* (LIMS-PS-3347).

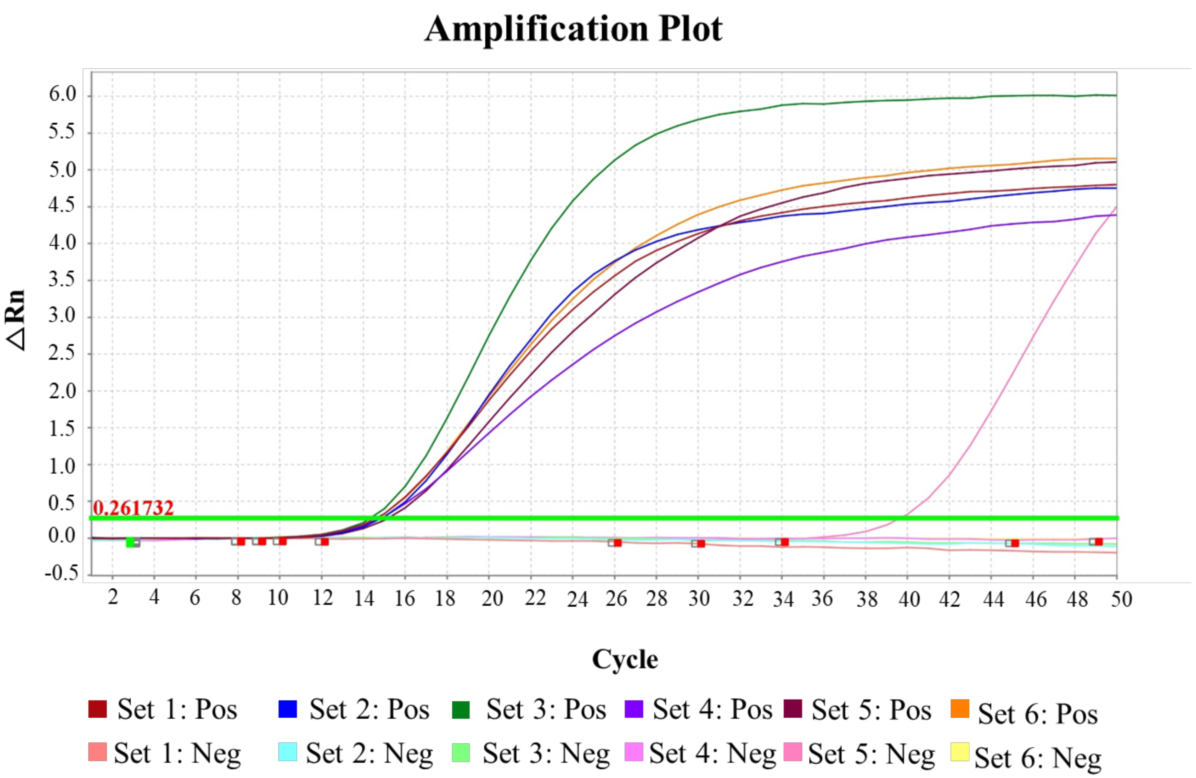


**Figure S4.** *G. polygramma* amplification plot showing the qPCR reactions performed using species-specific PCR primer sets and fluorescent probes (molecular marker sets) that were originally designed for the target species.

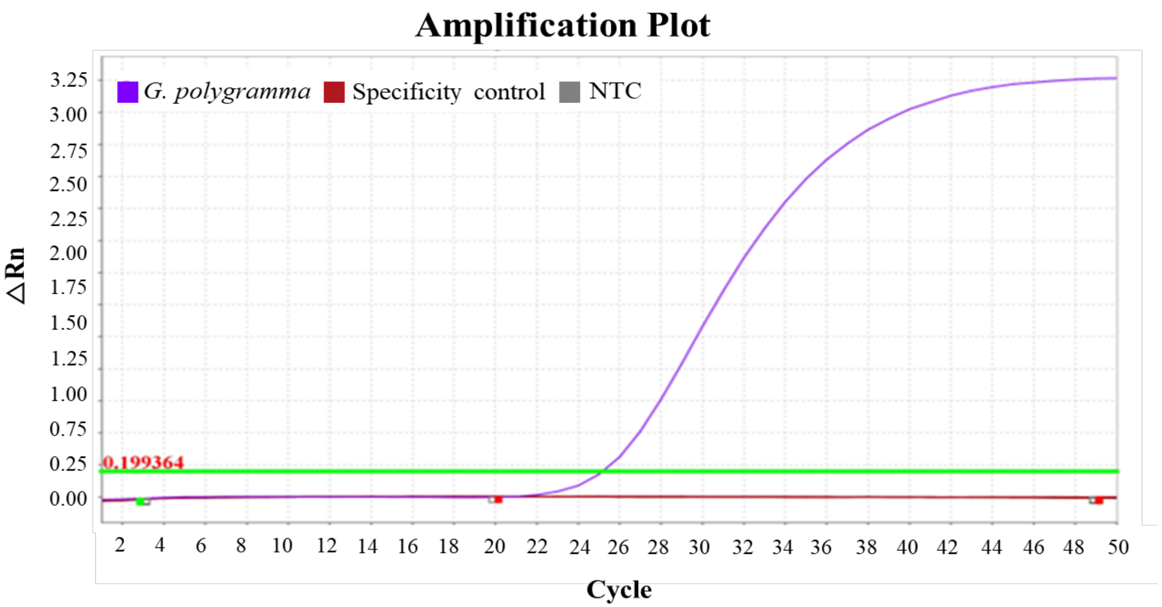


**Figure S5.** Specificity test of the molecular marker using 23 reference species and *G. polygramma* strain (LIMS-PS-3347).

**Supplementary Tables**

Supplementary Table S1. Sampling information and locations of the 25 seawater stations

| Station | Latitude [degrees_north] | Longitude [degrees_east] | Depth(m) |
|---|---|---|---|
| J1 | 35.122 | 128.601 | 11.0 |
| J2 | 35.087 | 128.673 | 10.0 |
| J3 | 35.046 | 128.747 | 12.0 |
| J4 | 35.019 | 128.793 | 13.0 |
| J5 | 35.065 | 128.643 | 14.0 |
| J6 | 35.039 | 128.533 | 16.0 |
| J7 | 34.975 | 128.606 | 17.0 |
| 1 | 34.816 | 128.294 | 22.0 |
| 2 | 34.586 | 128.303 | 30.0 |
| 3 | 34.805 | 128.133 | 17.0 |
| 4 | 34.687 | 128.133 | 20.0 |
| 5 | 34.576 | 128.137 | 30.0 |
| 6 | 34.550 | 128.023 | 22.0 |
| 7 | 34.577 | 127.842 | 10.0 |
| 8 | 34.432 | 127.665 | 20.0 |
| 9 | 34.475 | 127.553 | 24.0 |
| 10 | 34.320 | 127.720 | 34.0 |
| 11 | 34.385 | 127.500 | 24.0 |
| 12 | 34.145 | 127.500 | 38.0 |
| 13 | 34.348 | 127.167 | 27.0 |
| 14 | 34.240 | 127.165 | 23.0 |
| 15 | 34.238 | 126.950 | 27.0 |
| 16 | 34.109 | 126.958 | 45.0 |
| 17 | 34.258 | 126.794 | 47.0 |
| 18 | 34.117 | 126.789 | 33.0 |

Supplementary Table S2. List of non-target phytoplankton species used for assay specificity testing

| No. | Species name | Phylum | Class | Order | Family | Genus |
|---|---|---|---|---|---|---|
| 1 | *Chlamydomonas hedleyi* | Chlorophyta | Chlorophyceae | Chlamydomonadales | Chlamydomonadaceae | *Chlamydomonas* |
| 2 | *Dunaliella salina* | Chlorophyta | Chlorophyceae | Chlamydomonadales | Dunaliellaceae | *Dunaliella* |
| 3 | *Graesiella emersonii* | Chlorophyta | Chlorophyceae | Chlamydomonadales | Chlamydomonadales familia incertae sedis | *Graesiella* |
| 4 | *Tetradesmus obliquus* | Chlorophyta | Chlorophyceae | Sphaeropleales | Scenedesmaceae | *Tetradesmus* |
| 5 | *Tetraselmis suecica* | Chlorophyta | Chlorodendrophyceae | Chlorodendrales | Chlorodendraceae | *Tetraselmis* |
| 6 | *Micractinium singularis* | Chlorophyta | Trebouxiophyceae | Chlorellales | Chlorellaceae | *Micractinium* |
| 7 | *Chlorella vulgaris* | Chlorophyta | Trebouxiophyceae | Chlorellales | Chlorellaceae | *Chlorella* |
| 8 | *Pseudochlorella pringsheimii* | Chlorophyta | Trebouxiophyceae | Prasiolales | Koliellaceae | *Pseudochlorella* |
| 9 | *Synechococcus rubescens* | Cyanobacteria | Cyanophyceae | Synechococcales | Synechococcaceae | *Synechococcus* |
| 10 | *Gloeothece tepidariorum* | Cyanobacteria | Cyanophyceae | Chroococcales | Microcystaceae | *Gloeothece* |
| 11 | *Attheya longicornis* | Heterokontophyta | Mediophyceae | Biddulphiales | Attheyaceae | *Attheya* |
| 12 | *Minutocellus polymorphus* | Heterokontophyta | Mediophyceae | Cymatosirales | Cymatosiraceae | *Minutocellulus* |
| 13 | *Chaetoceros curvisetus* | Heterokontophyta | Mediophyceae | Chaetocerotales | Chaetocerotaceae | *Chaetoceros* |
| 14 | *Nannochloropsis oceanica* | Heterokontophyta | Eustigmatophyceae | Eustigmatales | Monodopsidaceae | *Nannochloropsis* |
| 15 | *Tisochrysis lutea* | Haptophyta | Coccolithophyceae | Isochrysidales | Isochrysidaceae | *Tisochrysis* |
| 16 | *Prymnesium parvum* | Haptophyta | Coccolithophyceae | Prymnesiales | Prymnesiaceae | *Prymnesium* |
| 17 | *Diacronema viridis* | Haptophyta | Pavlovophyceae | Pavlovales | Pavlovaceae | *Diacronema* |
| 18 | *Phaeodactylum tricornutum* | Heterokontophyta | Bacillariophyceae | Naviculales | Phaeodactylaceae | *Phaeodactylum* |
| 19 | *Effrenium voratum* | Dinoflagellata | Dinophyceae | Suessiales | Symbiodiniaceae | *Effrenium* |
| 20 | *Amphidinium carterae* | Dinoflagellata | Dinophyceae | Amphidiniales | Amphidiniaceae | *Amphidinium* |
| 21 | *Gymnodinium catenatum* | Dinoflagellata | Dinophyceae | Gymnodiniales | Gymnodiniaceae | *Gymnodinium* |
| 22 | *Alexandrium catenella* | Dinoflagellata | Dinophyceae | Gonyaulacales | Pyrocystaceae | *Alexandrium* |
| 23 | *Alexandrium pacificum* | Dinoflagellata | Dinophyceae | Gonyaulacales | Pyrocystaceae | *Alexandrium* |

Supplementary Table S3. Global occurrence patterns of *G. polygramma* and associated impacts on marine organism mortality in diverse geographical regions

| Area | Period | Temperature (°C) | Salilnity | Number of cells (cells $L^{-1}$) | Mortality | Source |
|---|---|---|---|---|---|---|
| South Africa, False Bay | Mar., Apr. 1962 | 17 ~ 22.1°C | 34-35 | - | Fish, invertebrates | Grindley et al., 1964 |
| Japan, Uwajima Bay | Aug., Nov. 1994 | 21.3 ~ 29.4 °C | - | $6.8 \times 10^7$ | Fish, shellfish | Koizumi et al., 1996 |
| Belize, Douglas Cay | May 1995, 1996 | - | - | $3.5 \times 10^6$ (1995), $1.8 \times 10^6$ (1996) | - | Morton & Villareal, 1998 |
| Korea, Yeosu | Aug. 2004 | 19.2–23 | 31.9–33.0 | $2 \times 10^6$ to $4 \times 10^6$ | - | Cho, 2005 |
| Mexico, Gulf of California | October. 2004 | 29 ° | 35.5 | $4.1\times 10^6$ | - | Gárate-Lizárraga et al., 2006 |
| Korea, Southern Coast | Aug, 2009 | 22-26 | 29.0–32.5 | $6.5 \times 10^6$ to $12.5 \times 10^6$ | - | Cho, 2011 |
| Oman, Southern Coast | - | 25–32 | - | - | Fish | Al Gheilani et al., 2011 |
| South Africa, Southern Benguela | 2011 | 16–18°C | 35.8–362 | $2.2 \times 10^5$ | Fish | Van der Lingen et al., 2016 |
| Caspian Sea | June 2010,<br>Aug 2013 | - | - | $4.5 \times 10^5$<br>$1.8 \times 10^5$ to $3.5 \times 10^5$ | - | Pautova et al., 2017 |
| India, Mangalore | Oct. 2008 | 29.55 | 34.0–34.2 | $5 \times 10^8$ | Zooplankton | Padmakumar et al., 2018 |
| India, Kochi | Nov. 2016 | 27.8–31.5 | 34.1–35.4 | $4.9 \times 10^6$ | - | Kumar et al., 2020 |
| Oman, Northern Oman Sea | Oct. 2017 | 28–30 | 36 | $15 \times 10^6$ | - | Dolatabadi F et al., 2021 |
| Korea, Yeosu | Aug.2021 | 26-28 | 30-31 | $4.0\times 10^2$ | - | This study |

Supplementary Table S4. Composition of standard PCR mixture

| Reagent | Amount |
|---|---|
| Genomic DNA (10 ng/µl) | 2 µl |
| Forward primer (5 pmol/µl) | 1 µl |
| Reverse primer (5 pmol/µl) | 1 µl |
| PCR-grade water | 16 µl |
| Total | 20 µl |

Supplementary Table S5. Composition of the qPCR mixture for the detection of *G. polygramma*

| Reagent | Amount |
|---|---|
| qPCR Master Mix | 10 μL |
| gDNA | 2 μL |
| Forward primer (5 pmol/μL) | 1 μL |
| Reverse primer (5 pmol/ μL) | 1 μL |
| Taqman probe (5 pmol/ μL) | 0.5 μL |
| CXR reference dye | 0.2 μL |
| Nuclease-free water | 5.3 μL |
| Total | 20.00 μL |

Supplementary Table S6. PCR amplification results of *G. polygramma* using species-specific primer–probe sets

| Set no. | Primer set | qPCR amplification | Probe | Remark |
|---|---|---|---|---|
| 1 | Gpo-28S-0430f/0625r | Y | Gpo-28S-0587p | |
| 2 | Gpo-28S-0430f/0732r | Y | Gpo-28S-0587p | |
| 3 | Gpo-28S-0521f/0625r | Y | Gpo-28S-0587p | |
| 4 | Gpo-28S-0521f/0732r | Y | Gpo-28S-0587p | |
| 5 | Gpo-28S-0535f/0625r | Y | Gpo-28S-0587p | NTC Dected |
| 6 | Gpo-28S-0535f/0732r | Y | Gpo-28S-0587p | |
